# Pulling Off Stable Economic System Adhering Carbon Emissions, Urban Development and Sustainable Development Values

**DOI:** 10.3389/fpubh.2022.814656

**Published:** 2022-02-10

**Authors:** Cen Cai, Ran Qiu, Yongqian Tu

**Affiliations:** ^1^School of Finance, Shanghai University of Finance and Economics, Shanghai, China; ^2^Chengdu Santai Intelligent Technology Co., Ltd., Chengdu, Sichuan, China; ^3^Research Fellow of National Academy of Development and Strategies, Renmin University of China, Beijing, China

**Keywords:** sustainable development values, economic growth, urban development, carbon emissions, CO_2_

## Abstract

In today's world, sustainability has become a major concern. The current study attempts to look into the impact of six sustainable development values on economic growth and emissions of carbon footprints: freedom, equality, solidarity, tolerance, respect for nature, and shared mobility. The moderating importance of urban development in regulating the relationship of economic growth and carbon footprints has also been investigated in this study. The structural equation modeling (SEM) analysis technique was utilized to confirm pathways and verify path modeling in this work. Overall, 302 responses were selected for data analysis, and data were collected using a self-administered questionnaire with a random sampling technique. The study found that two of the six sustainable development values (tolerance and solidarity) have a beneficial impact on a country's economic growth. However, freedom, equality, respect for nature, and shared mobility are not indicators of a country's economic progress. Similarly, economic expansion helps to mediate the relationship between tolerance, solidarity, and carbon emissions. Although urban growth did not play a moderating role, it has a positive impact on carbon emissions. The current study suggests various implications for long-term development to improve an economy's or country's economic performance. Furthermore, complete emphasis must be paid to sustainable development values, which are more closely linked to economic growth but also help cut carbon emissions. Future research should look into the current model in other countries.

## Introduction

A country's economic performance can be directly related to sustainable development and is suggested by many researchers in past (1, 2). Similarly, carbon emissions plays an important role in the changing climate. Though, most of the times economic growth contributes a lot in carbon emission but there is a strong need to eliminate or reduce the association between economic growth and carbon emission. This could easily lead to green economy which is beneficial for the coming generations. In past, there have been less focus on finding out the sustainable development values which could lead to economic growth along with having positive impact on carbon emissions. This study focused on finding the relationship between these values, economic growth and carbon emissions. It is supposed that urban development could also lead to increased carbon emissions alongside the economic growth so, its regulating or moderating role between the two was also studied in this research.

Humans have a similar aim of sustainable development. So far, various definitions of sustainable development have been proposed, with the following being the most widely accepted: Sustainable development is defined as development that satisfies current demands without jeopardizing future generations' capacity to meet their own (3). The watchword for international assistance organizations, the vocabulary of development planners, the focus of conferences and research publications, as well as the slogan of development and environmental activists, sustainable development has become a ubiquitous development paradigm. The widespread attention has been sought by the idea that other concepts of development have not, and this type of development is the dominant one and seems to be on the right track for longer periods of time (4). Improving the quality of the environment is one of the most important conditions for achieving long-term development.

China has made considerable headway in environmental preservation in recent years, but air pollution remains a major barrier for long-term growth (5). Moreover, it is evident that it is a difficult task to change some of the human value for achieving the sustainable development (6, 7). The working relation of societies, individuals and organizations can be evaluated by the dedicated attitudes, defining the targets of the individuals involved. Values, in general, are ideas about desired targets which govern the choices as well as the appraisal for people, events and the behaviors ranked according to their relative significance. The general assembly of the United Nations has the recognition for the importance of modifications the choices of sustainable development values set by the people for directing the behaviors toward larger perspective of sustainable development worldwide. The General Assembly declared some basic values set which were equally important for relations among the nations in this era. The importance of these values in relations of the nations can be understood by the role of policy making and implementation, activities of the organizations and behaviors of the concerned persons. This is the most important task in the current era and will certainly have a great impact on living quality of the people globally (7, 8). This would also impact the conservation of the nature and social values.

The United Nations in their manifesto for the century defined certain fundamental values of the sustainable development such as solidarity, freedom, tolerance, equality, sharing of responsibility, and nature respect. Considering the significance of the relativity, there is not much understanding for the concept of sustainability norms, their nature and their role in aiding or impeding long-term development (9). Globally, GDP is used for metering the longevity of sustainable development along with the economic growth of the region. Yet, it could be understood that sustainable development and growth in the economy have certain distinct features, raising questions about whether GDP could be used to measure the sustainable development and the growth in economy and would be accepted? After the WWII GDP, became the primary source of measuring tool, when rapid economic expansion was critical for maintaining stable international relations. Because it was an excellent method to give an insight about exchange rate of consumption of energy, services and the goods and development of the capital as an indicator accomplishing its function. However, without taking into account the value of capital whether it be social, human or natural one, growth in the GDP growth raises disparity in the income for a certain period of time and a tipping point can be approached (10).

The Earth Charter expresses sustainable development principles, but certain specific values termed as the basic values of the sustainable development were defined and given in the manifesto for the century (11). Our study on economic growth and carbon emissions is focused on these core ideas. These variables provide the basis for assessing the role of sustainable development on growth in economy and carbon footprints. Among them freedom of the people's sustainability values were given the top priority which is elaborated as genders of women and men having the right of living with dignity and bring up their children without fear of violence, oppression, or injustice. These rights are best protected through democratic governments elected by the citizen of the country according to the will of people. The other significant value is equality which defines the sustainability value as no one, no nation, should be denied the chance to profit from growth.

Women's and men's equal rights and opportunities must be guaranteed. Solidarity is among the other core contributors as global issues should be addressed so diligently that there is a proper distribution of the costs or the burdens which is in accordance to the standards of fairness and principles of justice. The people who suffer the most or achieve the lowest profits are entitled to assistance from those who benefit the most. Similarly, tolerance has a great potential to offer toward the sustainable development contributing the economic growth as human beings must appreciate one another in all of their religious, cultural, and linguistic variety. Differences within and across communities should not be feared or suppressed, but rather appreciated as a valuable human asset. All ethnicities should proactively cultivate a culture of peace and discussion. Same is the case with respect for nature in which every living creatures and environmental assets must be managed with caution. This should be done according to the sustainable development principles. So, accordingly the nature's vast resources be maintained and passed down to future generations.

In order to ensure our prospective wellbeing and that of our successors, we should alter our unsustainable patterns of consumption. Last but not the least is the shared responsibility which should comply with the sustainable development according to which there is a responsibility of maintaining the growth socially and economically, alongside managing the risks which are associated to peace and security of international communities and these responsibilities should be shared between all the stakeholder nations in a multilateral manner. The United Nations, as the world's most universal and representative body, must take the lead. All these value variables are defined according to (9) in order to develop scales for measuring the values that underline sustainable development. The term “carbon footprint” comes from the phrase “ecological footprint,” which refers to the entire quantity of gases produced due to greenhouse effects released to the atmosphere by the human activities of production and consumption. This also refers to the study of manufacturing and distribution carbon emission processes that are directly or indirectly linked to human activities. Carbon footprint has become a new subject of research throughout the world as a revolutionary means of quantifying the amount of carbon generated by human activities (12).

The majority of existing literatures are focused with carbon footprint computation and evaluation, carbon footprint reduction approaches, carbon footprint modeling, and comparisons of carbon footprints from various industries. There is very little known for computing the impact of sustainable development values on carbon footprints so, to check the impact this study was designed. Considerable work has been carried out in past on the relationship of sustainable development of the agriculture sector in regards to the carbon emissions as an idea of sustainable development of this segment and worldwide economy is significantly important. Around 30% of worldwide energy consumption is consumed by the agri-food industry. It also contributes to the release of a considerable amount of greenhouse gases into the atmosphere. Food items' carbon footprint is governed by a variety of factors related to their manufacture. Animal-based foods are more hazardous and have larger carbon footprints than plant-based foods. The use of renewable energy sources, as well as the avoidance of chemical fertilizers and plant protection products, can help to reduce carbon emissions (13).

Along with sustainable development role in economic growth and carbon footprints, urban development could also play a role in regulating the outcomes. Since, urban development is an old concept so, sustainable urban development can be considered in determining its role. The amount of urbanization in a country is a measure of its success in raising the national quality of living, as well as an indicator of the country's economic and overall strength. Cities use limited natural resources to establish a social economy and constantly improve living conditions as they urbanize. However, as urbanization processes proceed, environmental issues connected with urban economic development emerge, such as urban heat islands, air pollution, and resource exhaustion. Consequently, good urban development is a topic of research, and sustainability is rapidly becoming a focus in urban development (14, 15). As urbanization accelerates in both developed and developing countries, urban development that improves urban operations and creates opportunities for economic development is required to provide all residents with lifelines, transportation, the environment, education, medical care, housing, employment, and other amenities (16, 17).

The provision of equal services and a safe, secure urban infrastructure will be critical in increasing the value of cities for inhabitants, business people, and visitors from other countries. So, moderating role of urban development cannot be ignored in between economic growth and carbon footprint (18). This research was focused on certain objectives such as sustainable development values, their role toward economic growth and carbon footprints and analysis of moderating role of urban development.

This study has been structured as first section describe about the introduction and second section describe about the literature review. The methodology and data collection has been described in the third section and fourth section explains about the study analysis and results. The discussion, concluding remarks and study limitation has been added in the last section.

## Review of Literature

### Foreign Direct Investment Theories and Their Role in Economic Growth and Carbon Footprint

Over the years, the link between economic growth and foreign direct investment gotten tremendous attention. Despite, critical importance of FDI in economic growth, many policymakers are unaware of the theoretical relationship between FDI and economic growth. Theoretical research has shown that FDI is a significant contributor to the host country's economic growth. FDI affects economic growth in two ways as (1). Through technological spillovers, FDI can encourage the adoption of new technologies in the production process; and (2). Through knowledge transfers, both in terms of labor training and skill acquisition, as well as by introducing alternative management practices and better organizational arrangements (19).

The capital, manufacturing processes, skills of management, knowledge of the marketing, products and services, their advertisement, and company organizational procedures are all part of FDI. FDI is said to have significant economic benefits on host economies. In principle, according to the theory of Exogenous growth, FDI may help a host country's economy grow by accumulating cash, introducing new items, and introducing technology acquired from abroad (20). According to hypothesis of endogenous growth, Knowledge accumulation in the host nation can be increased through transferring talents. According to the endogenous growth hypothesis, it can also increase the stock of knowledge in the host nation through transferring talents. It was stressed that FDI contributes significantly to the host country's growth of economy growth by generating investable funds and facilitating technical spill outs (21).

Similarly, as per foreign direct investment (FDI) theory, investors are the source of transferring pollution of the carbon emissions to borders across from the high emitting regions to the countries which are low-income states. Therefore, they are the responsible of transfer of emissions to the other countries. Many sectors of the export which use energy the most due to which the carbon emissions significantly increase, become the sites of production for countries which invest their capital hugely and ultimately chip in the reasons of pollution. It is due the fact that prices for energy are lesser in the regions and the laws for regulation of such business are nearly meager as compared to the countries with developed economies (22). Furthermore, there is an impact of technology transfer on the cycles of carbon emissions due to the FDI. This can undoubtedly improve the technology in the region but with an impact of carbon footprints in the host country. Consequently, FDI plays an important role in carbon emissions of the host countries.

Another supportive theory for the current study was carbon footprint theory according to which, there is currently no commonly acknowledged and clear definition of a carbon footprint. However, the concept of a footprint does exist. The carbon footprint is a summation quantity of carbon dioxide emissions internally and externally induced by an activity or generated during the life phases of a product, as proposed by (23). The carbon footprint, on the other hand, is a measurement of carbon dioxide emissions (24).

### Impact of Sustainable Development Values on Economic Growth

The virtue of association among progress in economy and sustainable development value of freedom has been the subject of several research. Scholars sought to address the same topic having used the range of methodologies of econometrics and indices of sustainable freedom to address the question that: can sustainable freedom play a role in contribution to growth of economy? Some different researches in past also probed in to identify the aspects of sustainable freedom contributing toward growth in economies. Two different schools of thoughts have been evolved in this regard. Among them, the first one was focusing the causative relationship among indices of growth in economies and sustainable freedom (25). The nature of the link between the level of democracies, governmental, personal, socioeconomic freedom, and growth was the topic of numerous research (26). The results of these research were mixed. According to quantitative or econometric technique utilized by the researchers, some of the findings revealed that no substantial association existed, while others revealed the impact of certain characteristics of sustainable freedom on growth in economy.

Some academics looked at the effects of freedom on growth of the economies of various nations at certain socio-economic levels. For the first decade of this century, a panel study was conducted by them. They took 94 countries as a sample for the analysis. According to their findings freedom at economic level showed positive and statistically significant link with growth in economy among all the participating countries. This was regardless of their level of income. Others looked at the connection among freedom at economic level and the status of 30 members economies of OECD countries, focusing on 4 years between 3 and 7 of the first decade of the century. Economic independence has a favorable influence on GDP levels, according to their panel research (27). In certain situations, the influence of economic freedom on economic development was determined using different sets of nations and criteria for grouping countries.

Inequality among the genders has been a hot debate among social economic researchers and other scientific communities. Gender equality is important globally, according to the United Nations; yet, inequality of the gender is even more devastating among few nations, particularly in the continents of Asia and Africa. Gender inequality, according to researchers, impedes economic progress for a variety of reasons. Gender disparity and social hurdles to employment for all socio-economic groups are created by the economic and social framework, which impedes long-term economic progress (28). Researchers have shown that disparity among the genders has a detrimental influence on economic growth depending on the ability to have education and other contributing factors. According to the study, education of the female gender can lower levels of the fertility with the rise in level of educational of coming generations. Women with greater education have more work prospects, according to these studies, which has a direct influence on economic growth (29, 30). This proves the role of equality for the economic growth of any region. Another important problem impacted by gender inequality is health (31). In the literature and in conferences, the connection between access to healthcare and promotion of health and growth in economy have been explored. Human capital is essential for economic progress and is a key component in determining long-term success. Human capital investment techniques include increasing education and enhancing health and healthcare access. According to studies, a clear link was found between growth in economy and health (32).

For the last several years, politicians, scholars, and practitioners throughout the world have been more interested in the potential of the social and solidarity economy (SSE) to address today's key concerns, such as hunger, unemployment, discrimination, social isolation, and climate change. SSE strives to develop values for the people and local communities depending upon the collaboration, principles of fairness, solidarity and inclusiveness. Its efforts are mainly depending upon satisfying needs of the community and fostering a sustainable and inclusive society which provide empowerment to the segments of vulnerable people. In the last decade, the Korean government has built a number of supporting legislative frameworks and regulations for SSE and resultantly, a huge number of organizations and companies have sprung up across the nation. Alongside, increasing agreement could be seen that in Korea segments of SEOEs are well-deployed to achieve the goals of sustainable development, it is less apparent that how good they are performing regarding practice. The research looked at economic, social, environmental effects of SSE in South Korea, as well as how these effects relate to the SDGs (33). A number of studies also revealed that solidarity could be a leading link toward sustainable development and the economic growth of the less developed regions. Tolerance for ambiguity is one of the dimensions of sustainable development, and it refers to people's ability to adjust and conform to new and unclear situations. As a result, the higher each dimension's scale, the more people see different cultures, values, and behaviors. Also, “the capacity to communicate successfully in cross-cultural circumstances and to react appropriately in a range of cultural contexts” can be defined as intercultural competence. Tolerance, on the other hand, is described as an open-minded and courteous attitude toward variety, regardless of people's ethnic, social, or preferred lifestyles. To some extent, this concept corresponds to the findings of other scholars. As a result, variables that describe a person's sentiments toward individuals from other social groups may be used to measure tolerance (34).

This approach has been extended to various situations in a closely related literature. Researchers looked at China and discovered a link between tolerance and GDP per capita, as assessed by the proportion of a region's population who come from other regions of the nation (showing openness) (35). Consequently, the study attempts to integrate and evaluate the influence of social tolerance on economic development, given that tolerance and variety serve comparable functions in recruiting human capital. This is evidenced by the fact that tolerance fosters a climate conducive to the development of human capital, which is essential for economic progress. This is demonstrated by the fact that highly educated people are drawn to regions that are known for their inclusivity and diversity (36).

Respect for nature is above everything for the economic growth as in past, uncontrolled emissions have had a very bad impact on the climate which is extremely bad for us and the coming generations. Researchers utilized this integrative idea to look at how energy reserves needed to create labor and important resources like minerals deteriorate over time. Anomalies in the geology offering low-entropy energy stocks an access permitted current levels of economic expansion (37), and depletion in their level will raise the level of energy and expenditure which is required to get access to less accessible quantities. This kind of thermodynamic deterioration has not been taken into account in contemporary economic models, and market-based regulation was built only by neglecting the biosphere's physical and biological boundaries (38, 39).

Free-trade proponents, those who believe that technology will address the difficulties coming forward, and the ones who get heavy profits by the overuse of resources are three society divisions that will likely fight a shift in viewpoint based on respecting natural boundaries as individuals and economic sectors. The first two supply the gasoline for the race ahead, while the third provides the tools to complete it. These performers profit from their ability to tap into people's need for novelty, material possessions, social status, and identity. They see people as self-centered individuals who compete for wealth and power in social relationships (40). Daly pioneered the notions of sustainability and circular economies, which depend on recycling of the resources. Recycling and increased energy efficiency, on the other hand, may delay but not stop resource degradation. Furthermore, as Jevons demonstrated over 150 years ago, improvements in energy efficiency do not always result in decreased net consumption and might instead stimulate demand by decreasing prices (41). The impact of the sharing economy on the environment is largely unknown. It does not automatically result in lower resource usage and improved environmental outcomes. It is untrue to say that the sharing economy is necessarily sustainable. Some experts even claim that the sharing economy is only a notion used by entrepreneurs for their own financial gain rather than for the benefit of others, and that it is unlikely to create a sustainable change. The sharing economy has changed the way people do business, spawned new organizational structures, and influenced consumer culture. The sharing economy, which began as a movement promising an inclusive economy, democratic and more sustainable, is now topic of the heated discussion due to its influence on problems such as privacy, discrimination, worker rights, and regulation (42). Based on the theoretical and the literature support on sustainable development fundamental values, following hypotheses were proposed.

***H***_**1**_*: Freedom significantly predicts economic growth*.
***H***_**2**_*: Equality significantly predicts economic growth*.
***H***_**3**_*: Solidarity significantly predicts economic growth*.
***H***_**4**_*: Tolerance significantly predicts economic growth*.
***H***_**5**_*: Respect for nature significantly predicts economic growth*.
***H***_**6**_*: Shared responsibility significantly predict economic growth*.

### Impact of Economic Growth on Carbon Emissions

Economic growth is a key metric for assessing a country's or region's economic development. Whenever the countries having a lower growth in economies consume limited resources of energy, their carbon gas emissions are modest. Nevertheless, as industrialization progresses, more fossil energy is required, environmental degradation increases, and carbon emissions increase. When development in economic levels attains a certain value, amount of carbon emissions and pollutants steadily decreases (43). The literature supported the formulation of the following hypothesis.

***H***_**7**_*: Economic growth has a significant impact on carbon emissions*.

### Impact of Urban Development

Urbanization has a significant impact on carbon emissions. First, the amount of urbanization in a nation is intimately linked to growth in economy. The greater scope of urban development resulted greater increase in carbon emissions. Improvements in urbanization levels, on the other hand, can reduce carbon emissions and so enable low-carbon growth. Sun discovered that expanding the scope of urbanization promotes improved carbon emission efficiency in the early phases of urban growth (44). When the amount of urbanization reaches a crucial threshold, economic growth lags behind the pace of increase in carbon emissions. Second, the building of infrastructure during the urbanization process necessitates a considerable amount of energy resources, resulting in a rise in carbon footprints. With the development of new structures and preservations of the existing available land, Zhou discovered that spatial urbanization was positively related with carbon emissions. Third, as a result of urbanization, many people migrate from rural to urban regions, resulting in a significant rise in carbon emissions (45–48). Urban development plays an integral role in economic growth and sustainable development ultimately contributing toward carbon emissions so, it was necessary to analyze its regulating and moderating role between economic growth and carbon emission. The testing of urban development as a moderator was supported by the previous studies such as (49) in which moderation of the urban development was tested on the EKC which is environmental Kuznets curve. The study's main goal was to look at the long term effects of growth of the economy, openness of trading, urban development and energy consumption on environmental degradation in the country of Turkey, as well as the causative relation between the variables taking urban development as moderator from 1960 to 2016. For the example of Turkey, the results of the long-run estimators demonstrated that the existence of a moderating influence of urban development on indicators of carbon emissions was validated. Furthermore, while openness of the trading, urban development and energy consumption were relevant to the projecting carbon footprints, the moderation of urban development led to carbon emissions in the short term, according to the causality tests. All this literature supported the hypothesis development as given below.

***H***_**8**_*: Urban development has a significant role toward carbon emissions*.
***H***_**9**_*: Urban development significantly moderates the linkage between economic growth and carbon emissions*.

### Impact of Economic Growth as Mediator Between Sustainable Development Values and Carbon Emission

Sustainable development is highly tied to socio-economic, human development, and socio-political aspects. Strong performance of the economy boosts money, particularly through investments of the capital in healthcare, education and infrastructure. Following the expansion of manufacturing and services, the link between development of the economy and altering levels of money and wealth demonstrates contribution of the natural capital has decreased, Alongside, the percentage of capital created and capital which is intangible has grown. Despite this, present economic paths are unsustainable for future generations due to the loss of numerous natural resources (50). Economic growth refers to the gradual expansion in the economy's productive capacity through time, resulting in higher levels of national production and revenue. It denotes an increase in output or an expansion of the economy. The rise in the value of an economy's commodities and services could be referred as growth in economy.

Growth of economy can be defined and measured as a percentage growth in output of real GDP. Growth is commonly assessed in real terms, or inflation-adjusted terms, to account for the effect of inflation on the price of the products and services produced. The rise in potential output, or production at “full employment,” as a result of higher aggregate demand or observed output is referred to as “economic growth” or “economic growth theory” in economics. Economic growth is defined as the annual percent change in national income, which includes all of the advantages and disadvantages associated with that level variable. People, on the other hand, prefer to assign a specific value to the yearly percentage change, maybe because it tells them what will happen to their paycheck (51). Economic growth has been studied as a strong link for sustainable development along with being a predictor of any economy.

Several factors had been contributing toward economic growth and reported in literature precisely but role of economic growth as a mediator among other players is very limited such as sustainable development values and carbon footprints. The fact is, there is a huge gap in literature in defining its role as a mediator. A study was conducted in past to analyze the mediating role of economic growth by (52). This research basically focused on patterns of carbon emissions and factors influencing in the country of China. Economic growth was a mediator of CO_2_ emissions when it came to energy consumption, foreign commerce, administration of the government and industrial structure. Hence, its contribution as mediator suggested to develop hypotheses testing mediating role of economic growth between sustainable development values and carbon emissions. So, following hypotheses were formulated and tested accordingly.

***H***_**10**_*: Economic growth plays a mediating role among sustainable development value of freedom and carbon emissions*.
***H***_**11**_*: Economic growth plays a mediating role among sustainable development value of equality and carbon emissions*.
***H***_**12**_*: Economic growth plays a mediating role among sustainable development value of solidarity and carbon emissions*.
***H***_**13**_*: Economic growth plays a mediating role among sustainable development value of Tolerance and carbon emissions*.
***H***_**14**_*: Economic growth plays a mediating role among sustainable development value of respect for nature and carbon emissions*.
***H***_**15**_*: Economic growth plays a mediating role among sustainable development value of shared responsibility and carbon emissions*.

**Figure 1:**
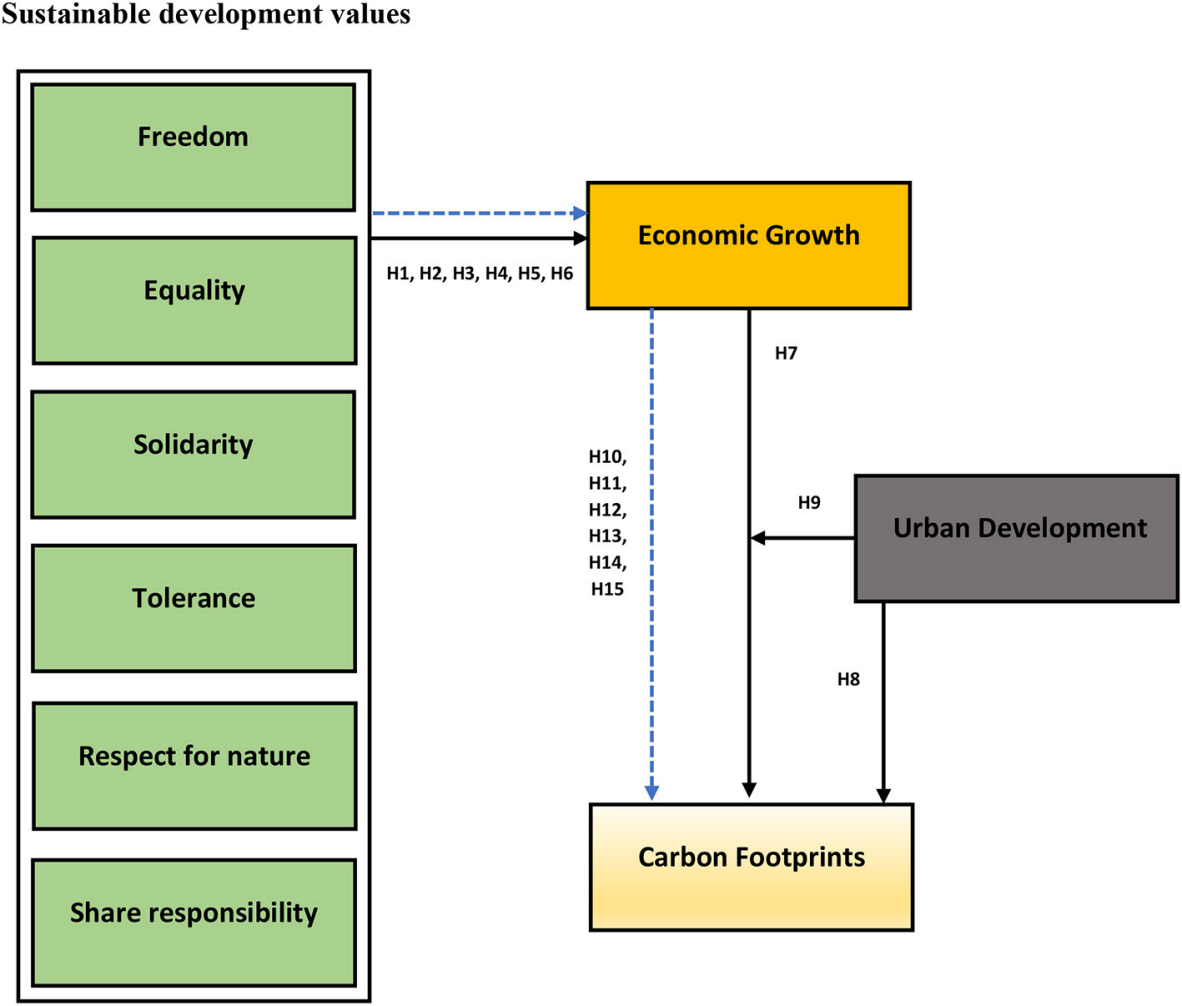
Conceptual framework.

Based on the literature and hypothesis, following framework has been established as show in (Figure 1).

## Research Methods

The positive philosophy supports the current subject of research. The study is survey-based research and because the data was collected at a single point in time hence it is cross-sectional nature research (53). The survey research provides cost-effective, reliable, and accurate estimates. In the survey, a self-administrated questionnaire was adopted to gather data. Where the unit of analysis was employees from small-medium industries. The sampling technique used in the paper was a random sampling technique where every participant in the population has an equal probability of being chosen (54). Therefore, it minimizes the bias of responses. The total sample size of the study was 302, however, 350 questionnaires were distributed among population 48 were screened out during the screening process. The data was collected using social media platforms and the questionnaire was designed on Google-Forms. The excel sheet of responses was generated from Google-Forms and then transferred into Smart-PLS for structural equation modeling (SEM).

### Instrument Development

The constructs in the current research model were measured through well-established research instruments. In total, there were 33 items for nine constructs. There were six independent variables where freedom, equality, solidarity, tolerance, respect for nature, and shared responsibility have three items for each however four for shared responsibility. The measurement scale for all sustainable values is taken from (9). The measurement scales for economic growth, urban development, and CO_2_ (carbon emission) were taken from (52). Where economic growth has three items, urban development also three items, and CO_2_ (eight items) (52). All items were modified merely to adjust them according to current settings however it has not changed the meaning of the respective statement. These statements were given on a 5-point Likert scale from strongly agree (5) to strongly disagree (1).

### Data Analysis Technique

The development of a conceptual model was examined using the Smart-PLS version 3.3.2 application, which used the Smart-Partial Least Square Structural Equation Modeling PLS-SEM. The method is divided into two parts: (i) measurement model evaluation and (ii) structural model evaluation. Previous research has recommended that these two processes be traded off utilizing a one-step technique. The measurement evaluation shows how all variables in the model are measured, whereas the structural model assessment identifies how variables in the model are related to each other. The measurement model estimation was based on the indicators, construct reliability, convergent and discriminant validity. However, the structural model tests the hypothesis based on the respective original sample (beta values), *p*-values, t-statistics, R^2^, and f^2^.

## Research Methods

### Demographic Details

The demographics of the respondent are classified into three categories age, gender, and qualification or education. Table 1 depicts the demographical summary of respondents where 52.98% were male respondents and 47.02 percent were female respondents. Moreover, the age of all respondents classified as 20 and less years (7.62%), 21–25 (15.56%), 26–30 (33.77%), 31–35 (9.93%), 36–40 (15.23%) and 41–45 (17.88%). The education of respondents were Bachelor and lower (33.77%), Master (26.16%), Doctorate 26.49%, Diploma, and others 13.58%.

**Table 1:** Demographics summary.

**Demographic**		**%**
**Gender**		%
Male	160	52.98%
Female	142	47.02%
**Age**		
20 and fewer years	23	7.62%
21–25	47	15.56%
26–30	102	33.77%
31–35	30	9.93%
36–40	46	15.23%
41–45	54	17.88%
**Education**		
Bachelor and lower	102	33.77%
Master	79	26.16%
Doctorate	80	26.49%
Diploma and others	41	13.58%
**Total**	302	

### Reliability Analysis and Measurement Model Assessment

**Figure 2:**
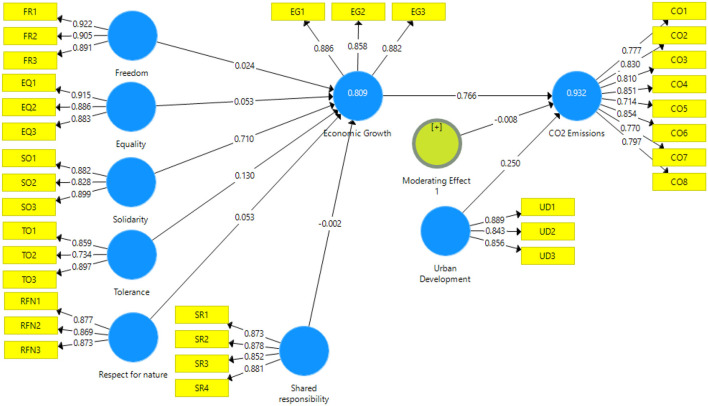
PLS-algorithm.

In the measurement and reliability analysis section authors have considered factor loadings, construct reliability, Cronbach alpha, average variance extracted (AVE), and Heterotrait-Monotrait (HTMT) ratio. The factor loadings are visible in (Figure 2) where all items in the respective construct have factor loadings higher than 0.70 thresholds (55, 56). Hence, the indicator reliability is maintained. The construct reliability was measured through constructs Cronbach alpha (α), and construct reliability (CR) and values for each construct is above 0.70 threshold point as CO_2_ emissions (0.920), economic growth (0.848), equality (0.875), freedom (0.892), respect for nature (0.844), shared responsibility (0.894), solidarity (0.840), tolerance (0.775), urban development (0.829). The construct reliability for CO_2_ emissions (0.935), economic growth (0.908), equality (0.923), freedom (0.932) respect for nature (0.906), shared responsibility (0.926), solidarity (0.903), tolerance (0.871), urban development (0.898). The convergent validity was measured through AVE, Table 2 illustrated that all AVE values are above 0.50 threshold point (57, 58). Hence, the result for convergent validity is satisfactory.

**Table 2:** Reliability convergent validity analysis.

**Constructs**	**Alpha**	**CR**	**(AVE)**
CO_2_ emissions	0.920	0.935	0.642
Economic growth	0.848	0.908	0.766
Equality	0.875	0.923	0.801
Freedom	0.892	0.932	0.821
Respect for nature	0.844	0.906	0.762
Shared responsibility	0.894	0.926	0.759
Solidarity	0.840	0.903	0.757
Tolerance	0.775	0.871	0.694
Urban development	0.829	0.898	0.745

The discriminant validity is measured through the HTMT ratio of correlation. The threshold point of the HTMT ratio is 0.85 (59, 60). The nearer the HTMT ratio close to zero (0) the better discriminant validity will become. The outcomes for the HTMT ratio are illustrated in (Table 3), where most of the values are below the threshold point however several values are above 0.85, which means the constructs in the current research model are lacking in discriminant validity hence more homogenous.

**Table 3:** HTMT ratio.

	**CO_**2**_**	**EG**	**EQ**	**FR**	**M1**	**RFN**	**SR**	**SO**	**TO**	**UD**
CO_2_									
EG	0.764									
EQ	0.924	0.885								
FR	0.238	0.244	0.213							
M1	0.527	0.481	0.650	0.142						
RFN	0.900	0.842	0.921	0.329	0.609					
SR	0.863	0.790	0.860	0.284	0.598	0.849				
SO	0.919	0.738	0.935	0.223	0.573	0.844	0.800			
TO	0.965	0.912	0.934	0.245	0.705	0.850	0.976	0.910		
UD	0.921	0.849	0.919	0.286	0.685	0.906	0.948	0.859	0.948	

*CO_2_, Carbon emissions; EG, Economic growth; EQ, Equality; M1, Moderating; RFN, Respect for nature; SR, Shared Responsibility; SO, Solidity; TO, Tolerance; UD, Urban Development*.

### Hypothesis Testing

**Figure 3:**
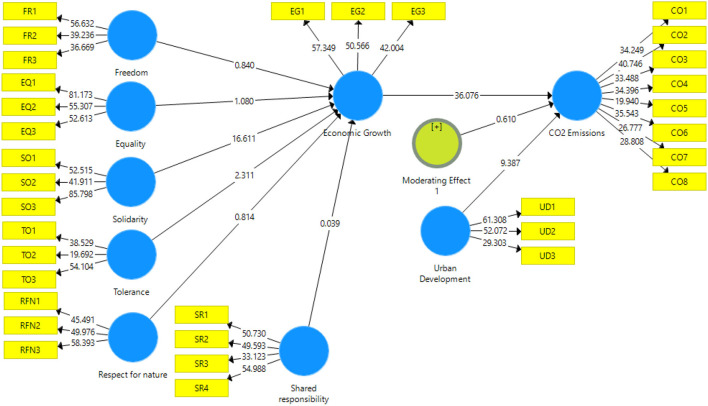
PLS-bootstrapping.

The structural model estimation was assessed to capture the casual linkage between constructs in the model. There were 15 hypotheses proposed in the current paper where eight were direct hypotheses, one moderating effect, and six mediating or indirect effects. The graphical representation is illustrated in Figure 3 and Table 4 for direct and indirect effects.

**Table 4:** Direct and indirect effects.

**H**	**Paths**	**O**	**M**	**SD**	**T Statistics**	**P-values**	**Results**	**R2**	**f2**
H1	FR → EG	0.024	0.028	0.028	0.840	0.401	Rejected	0.809	0.003
H2	EQ → EG	0.053	0.053	0.049	1.080	0.280	Rejected		0.004
H3	SL → EG	0.710	0.709	0.043	16.611	0.000	Accepted		0.883
H4	TO → EG	0.130	0.130	0.056	2.311	0.021	Accepted		0.021
H5	RFN → EF	0.053	0.054	0.065	0.814	0.416	Rejected		0.002
H6	SR → EG	−0.002	−0.002	0.058	0.039	0.969	Rejected		0.000
H7	EG → CO_2_	0.766	0.767	0.021	36.076	0.000	Accepted	0.932	4.272
H8	UD → CO_2_	0.250	0.248	0.027	9.387	0.000	Accepted		0.345
H9	M1 → CO_2_	−0.008	−0.009	0.013	0.610	0.542	Rejected		0.001
H10	FR → EG → CO_2_	0.018	0.021	0.021	0.842	0.400	Rejected		
H11	EQ → EG → CO_2_	0.041	0.041	0.038	1.073	0.284	Rejected		
H12	TO → EG → CO_2_	0.100	0.099	0.043	2.325	0.020	Accepted		
H13	RFN → EG → CO_2_	0.041	0.042	0.050	0.811	0.418	Rejected		
H14	SR → EG → CO_2_	−0.002	−0.001	0.045	0.038	0.969	Rejected		
H15	SL → EG → CO_2_	0.544	0.544	0.037	14.847	0.000	Accepted		

*H, Hypothesis; O, Original sample; M, Sample mean; SD, Standard deviation; CO_2_, Carbon emissions; EG, Economic growth; EQ, Equality; M1, Moderating; RFN, Respect for nature; SR, Shared Responsibility; SO, Solidity; TO, Tolerance; UD, Urban Development*.

First, four hypotheses were rejected where four sustainable development values such as freedom, equality, respect for nature and shared responsibility do not meaningfully predict the economic growth of a country because the linkage between these constructs is insignificant as *p value* = 0.401 , *t*−*statistics* = 0.840, *p value* = 0.280 , *t*−*statistics* = 1.080,*p value* = 0.416 , *t*−*statistics* = 0.814 and*p value* = 0.969 , *t*−*statistics* = 0.03. The outcome indicated that hypotheses (H_1_, H_2_, H_5_, and H_6_) were rejected. On the other hand, the outcomes *p value* = 0.000 , *t*−*statistics* = 16.611 and *p value* = 0.021 , *t*−*statistics* = 2.311confirmed that two of sustainability development values has positive significant impact on solidarity and tolerance. Hence, the hypotheses (H_3_ and H_4_) were accepted. In addition, economic growth has a positive impact on the CO_2_ (carbon emissions) *p value* = 0.000, *t*−*statistics* = 36.076, so hypothesis seven (H_7_) was accepted. Urban development also has positive impact on CO_2_ (carbon emissions) as *p value* = 0.000 , *t*−*statistics* = 9.387. The moderating effect of urban development on the linkage of economic growth and CO_2_ (carbon emissions) remain insignificant *p value* = 0.610, *t*−*statistics* = 0.542. Hence, hypothesis nine (H_9_) was rejected.

Six indirect mediating effects were proposed initially. Among those, economic growth does not play mediating or indirect role between four sustainable development values such as freedom, equality, respect for nature shared responsibility, and CO_2_ carbon emissions. The outputs were insignificant statistically hence the outputs (H_10_, H_11_, H_14_ and H_15_) showed an insignificant mediating role of economic growth *p value* = 0.400 , *t*−*statistics* = 0.842, *p value* = 0.284 , *t*−*statistics* = 1.073, *p value* = 0.020 , *t*−*statistics* = 2.325, *p value* = 0.418 , *t*−*statistics* = 0.811 respectively. On the other hand, the hypothesis (H_12_ and H_13_) were accepted as output *p value* = 0.969, *t*−*statistics* = 0.038 and *p value* = 0.000, *t*−*statistics* = 14.847 showed that economic growth plays a partial mediating role between tolerance, solidarity, and CO_2_ carbon emissions. The values for R^2^were 0.809 (80.9%) and 0.932 (93.2%) respectively which means a high impact of independent variables on the economic growth and carbon emissions. Likewise, effect size (f^2^) was also depicted in (Table 4) as it has three large effects, one medium and the other were small effect size.

## Discussion

This study moved around certain things such as role of fundamental values of sustainable development toward economic growth, carbon emissions, mediating role of economic growth among sustainable development values, carbon footprints and regulating role of urban development between growth in economy and carbon emissions. Along with these, impact of economic growth on carbon emission and role of urban development was also studied. Some of the sustainable development values showed significant impacts on economic growth but some showed disagreements with the reported literature and didn't contribute significantly toward economic growth. Our first four hypotheses were rejected including freedom, equality, solidarity and tolerance and their impacts on economic growth. Non-significant impact of freedom on economic growth is evident of lack of elaboration of the concept of freedom to the respondents as (25) and (61) got the nearly similar results in their research. Political freedom had non-significant impact on economic growth whereas our hypothesis a bit contradictory to their research as their economic freedom showed significance toward economic growth. Similarly, contradictory to the results of (62) and (28), our other hypothesis of impact of equality on economic growth was rejected. Again, the reason behind such results could be due to the ambiguity of the equality in aspects of the economic growth such as equality in education or health etc. Results for impacts of respect for nature and shared responsibility on economic growth were in accordance to the results of (37) and (42). These results are obtained due to the fact that some experts even claim that the sharing economy is only a notion used by entrepreneurs for their own financial gain rather than for the benefit of others, and that it is unlikely to create a sustainable change. Our 3rd and 4th hypotheses showed significant impact of solidarity and tolerance and are in accord to the results of (33) and (36). The possible reason for our results is the fact that concepts of solidarity and tolerance are providing help to the weak and community on a whole which ultimately contribute toward economic growth.

Discussing about mediating link of economic growth toward sustainable development values and carbon emission, it was evident that there was very limited or a few literature was available. The reason behind this lacking approach on evaluating the mediating role of economic growth is that economic growth itself is a vast field and is affected by many factors rather than mediating other variables. Our results showed partial significance about mediating role of economic growth between solidarity and tolerance and the results are in accordance to (52). The possible reason for such partial mediation is the fact that solidarity and tolerance are the tools of a weaker or common community which contribute the most toward economic growth.

Our hypothesis about the impact of growth in economy on increased carbon footprint emissions was significant in showing the strong link between the both players and the results are in accordance to many of the researchers of the past such as (52), but some researchers were of the view that after a certain achievement of economic growth there comes a steady decrease in carbon emissions and some declared that reduced emission of carbon footprint will result in loss of economic growth such as (63). The hypotheses about moderating role of urban development was not supported by the reported literature of (52), because urban development impacts the carbon footprints due to the onset of industry and carbon emission processes and don't moderate between economic growth and carbon emission but itself impacts the carbon footprints. which is in accordance to the results of (45) and (47). Ultimately, sustainable development values are of great importance for economic growth of any region.

## Conclusion

Sustainability has become an important concern in the current era. The primary predecessor economic, environmental, and social sustainability is referred for sustainable development. In 2015, the United Nations department of economic and social affairs released the 2030 Agenda for Sustainable Development (SDGs). Therefore, studying the role of sustainable development values in terms of enhancing economic growth and demolishing the carbon emissions footprints. The current research aims to investigate the impact of six fundamental values of sustainable development, tolerance, solidarity, freedom, share responsibility, and respect for nature on growth in economy and carbon footprints emissions. Moreover, it explored the mediation of economic growth and moderation of urban development. This study used the PLS-SEM analysis technique to confirm paths and test path modeling. Overall, 302 responses were considered for data analysis, and a random sampling technique was used while data collection through a self-administered questionnaire.

The findings of the current paper revealed that two of six sustainable development values (tolerance and solidarity) have a positive impact on the economic growth of a country. However, freedom, equality, respect for nature, and shared mobility do not predict the economic growth of a country. Likewise, economic growth plays a partial mediating role among tolerance, solidarity, and carbon emissions. The moderating role of urban development was insignificant however urban development positively influenced carbon emissions. The current research suggests several implications for sustainable development to improve economic performance within an economy or country. Moreover, full attention must be given to sustainable development values which are more related to economic growth however to reduce the carbon emission likewise. Future research is suggested to explore the current model in another country. Moreover, secondary data can give a clearer picture of the current findings. In this way, these findings can be generalized more appropriately and accurately.

## Limitation of the Study

The paper has few limitations. First, the study considered the human responses which were collected through questionnaire and the findings are reliant on those responses. Therefore, there may be bias in the findings hence scholars should explore current model on the basis of secondary data such as [64]. Secondary data can portray a clearer picture. Future research is suggested to explore the current model in another country. In this way, these findings can be generalized more appropriately and accurately. Finally, current research is cross-sectional hence more research is called to explore current model in longitudinal research.

## Data Availability Statement

The original contributions presented in the study are included in the article/supplementary material, further inquiries can be directed to the corresponding author.

## Ethics Statement

All subjects gave their informed consent for inclusion before they participated in the study. The study was conducted in accordance with the Declaration of Helsinki, and the protocol was approved by the Shanghai University of Finance and Economics (SUFC), China.

## Author Contributions

CC conceived and designed the concept literature review, data collection, and wrote the paper. RQ helped to provide technical support and contributed in analysis tools. YT has reviewed the work to improve the outcomes. All authors have read and agreed to the published version of the manuscript.

## Funding

This study was funded by National Key Research Program Research on Intelligent Disposal Technology of Multi-source Complaint Letters and Visits (2018YFC0831800).

## Conflict of Interest

RQ was employed by the company Chengdu Santai Intelligent Technology Co., Ltd. The remaining authors declare that the research was conducted in the absence of any commercial or financial relationships that could be construed as a potential conflict of interest.

## Publisher's Note

All claims expressed in this article are solely those of the authors and do not necessarily represent those of their affiliated organizations, or those of the publisher, the editors and the reviewers. Any product that may be evaluated in this article, or claim that may be made by its manufacturer, is not guaranteed or endorsed by the publisher.
